# Clinical value of miR-182-5p in lung squamous cell carcinoma: a study combining data from TCGA, GEO, and RT-qPCR validation

**DOI:** 10.1186/s12957-018-1378-6

**Published:** 2018-04-10

**Authors:** Jie Luo, Ke Shi, Shu-ya Yin, Rui-xue Tang, Wen-jie Chen, Lin-zhen Huang, Ting-qing Gan, Zheng-wen Cai, Gang Chen

**Affiliations:** 1 Department of Medical Oncology, The Second Affiliated Hospital of Guangxi Medical University, Nanning, Guangxi, Zhuang Autonomous Region China; 2 Department of Pathology, The First Affiliated Hospital of Guangxi Medical University, Nanning, Guangxi, Zhuang Autonomous Region China

**Keywords:** GEO, Lung squamous cell carcinoma, RT-qPCR, Target genes, TCGA

## Abstract

**Background:**

MiR-182-5p, as a member of miRNA family, can be detected in lung cancer and plays an important role in lung cancer. To explore the clinical value of miR-182-5p in lung squamous cell carcinoma (LUSC) and to unveil the molecular mechanism of LUSC.

**Methods:**

The clinical value of miR-182-5p in LUSC was investigated by collecting and calculating data from The Cancer Genome Atlas (TCGA) database, the Gene Expression Omnibus (GEO) database, and real-time quantitative polymerase chain reaction (RT-qPCR). Twelve prediction platforms were used to predict the target genes of miR-182-5p. Protein-protein interaction (PPI) networks and gene ontology (GO), and Kyoto Encyclopedia of Genes and Genomes (KEGG) analyses were used to explore the molecular mechanism of LUSC.

**Results:**

The expression of miR-182-5p was significantly over-expressed in LUSC than in non-cancerous tissues, as evidenced by various approaches, including the TCGA database, GEO microarrays, RT-qPCR, and a comprehensive meta-analysis of 501 LUSC cases and 148 non-cancerous cases. Furthermore, a total of 81 potential target genes were chosen from the union of predicted genes and the TCGA database. GO and KEGG analyses demonstrated that the target genes are involved in pathways related to biological processes. PPIs revealed the relationships between these genes, with EPAS1, PRKCE, NR3C1, and RHOB being located in the center of the PPI network.

**Conclusions:**

MiR-182-5p upregulation greatly contributes to LUSC and may serve as a biomarker in LUSC.

## Background

Lung cancer is a major cause of death associated with cancer, and its rate continues to increase [1, 2]. Non-small cell lung cancer (NSCLC) accounts for 80% of all lung cancer cases. Subgroups of NSCLC include lung adenocarcinoma, lung squamous cell carcinoma (LUSC), and lung large cell carcinoma. According to recent studies, most patients are diagnosed at an advanced stage, which leads to low cure rates. Only a minority of patients can be treated by surgery [1, 3]. Some patients are treated with chemotherapy, radiation therapy, and targeted therapy, but the 5-year survival rate remains low and many patients are at risk of recurrence [2–6]. The cure rate of lung cancer can be improved by early confirmation. At present, the diagnosis of lung cancer mainly depends on the pathological biopsy. Therefore, it is significant to explore the molecular mechanism of lung cancer and to improve its diagnosis and treatment [7].

MicroRNAs (miRNAs) are a type of endogenous non-coding RNA consisting of 19 to 24 nucleotides with regulatory functions [8–11]. miRNAs play important roles in biological processes. For example, miRNAs regulate the degradation of target mRNAs and deter their translation [8, 12]. Recent reports have shown that miRNAs are involved in the formation, development, and transformation of lung cancer [13–17]. Therefore, we can use miRNAs to detect, diagnose, and cure cancer [9, 13, 18–20].

MiR-182-5p, as a member of miRNAs family, can be detected in many cancers, for example, lung cancer, and the expression of miR-182-5p is upregulated [6, 21]. Several studies indicated that miR-182-5p acts as an onco-miR to enhance tumor cell proliferation [21–23]. However, previous studies have focused on particular aspects of miR-182-5p in LUSC and thus lacked a comprehensive description. The expression value of miR-182-5p was not shown in previous articles, which have often displayed *p* values of a statistical test. Therefore, data cannot be obtained. In this study, we analyzed 388 LUSC samples from The Cancer Genome Atlas (TCGA) and Gene Expression Omnibus (GEO) database to verify the clinical value of miR-182-5p in LUSC. Next, 23 clinical LUSC samples were used to further prove the clinical value of miR-182-5p. The PubMed, Wiley Online Library, EBSCO, Cochrane Central Register of Controlled Trials, Web of Science, Google Scholar, Ovid, EMBASE, and LILACS were also searched to obtain document sources. Furthermore, we used miRBase (http://www.mirbase.org/) to discern the target genes of miR-182-5p and investigated the enrichment pathways and target genes by KEGG pathway and GO enrichment analyses and protein-protein interaction (PPI) networks. On the basis of the previous literature, we combined more samples and using various methods to reduce the difference between the existing literatures. We hope this study provides comprehensive information on miR-182-5p for the occurrence and progression of LUSC.

## Methods

### MiR-182 expression in LUSC samples from TCGA database

TCGA database provides comprehensive cancer genomic datasets for researchers where data are available to search, download, and analyze. In this study, we searched TCGA database (https://cancergenome.nih.gov/) to examine miR-182 expression in LUSC tissues. We obtained the miRNA profiles of 338 LUSC tissues and 45 non-cancerous tissues together with the clinical info. Afterward, miR-182 expression was examined from the miRNA profiles. The extracted data were normalized and processed by log2 transformation. Subsequently, statistical analyses were performed to evaluate the miR-182 expression in LUSC tissues and the correlation between miR-182 expression and relevant clinical data. Additionally, to further analyze the overall survival of LUSC, a Kaplan-Meier curve was constructed using the median miR-182 expression value.

### MiR-182-5p expression in LUSC tissues from the GEO database

We mined the GEO database (http://www.ncbi.nlm.nih.gov/geo/) to obtain microarray profiles from LUSC samples using the following search terms: (cancer OR carcinoma OR adenocarcinoma OR tumour OR tumor OR malignanc* OR neoplas*) AND (lung OR pulmonary OR respiratory OR respiration OR aspiration OR bronchi OR bronchioles OR alveoli OR pneumocytes OR “air way”). The search results were then specified using the following filters: Series[Entry type], *Homo sapiens*[Organism]. The microarrays were selected according to the inclusion criteria as follows: miR-182 expression was examined in LUSC tissues and non-cancerous tissues. Microarrays were considered ineligible according to the following exclusion criteria: (1) microarrays did not meet the inclusion criteria; (2) the microarray profile did not include miR-182 expression; (3) the microarray only provided LUSC tissues without a control group; (4) an insufficient number of LUSC samples for analysis; and (5) microarrays used cell line samples. A total of seven datasets were obtained, namely, GSE16025, GSE25508, GSE29248, GSE47525, GSE19945, GSE51853, and GSE74190.

### Clinical samples

In our study, 23 formalin-fixed, paraffin-embedded LUSC tissues and their adjacent normal tissues were collected from the Pathology Department of the First Affiliated Hospital of Guangxi Medical University between January 2012 and February 2014. All samples were pathologically confirmed as LUSC by two independent pathologists (Z.-y.L. and G.C.). The study was approved by the Ethics Committee of the First Affiliated Hospital of Guangxi Medical University, and the clinical parameters of 23 patients were shown in Table 1.

**Table 1:** Clinical parameters of 23 LUSC patients

Clinicopathological parameter		*n*
Tissue	LUSC	23
	Non-cancer	23
Gender	Male	18
	Female	5
Age (years)	< 60	15
	≥ 60	8
Smoke	No	12
	Yes	11
Tumor size	≤ 3 cm	7
	> 3 cm	16
Vascular invasion	No	20
	Yes	3
TNM	I-II	10
	III-IV	13
Lymph node metastasis	No	11
	Yes	12
Pathological grading	II	16
	III	7

### RT-qPCR

To detect the expression of miR-182 in 23 pairs of samples, RT-qPCR was carried out on an Applied Biosystems PCR 7900 system. Total RNA was extracted and normalized as previously reported [24–28]. The expression levels of miR-182 were evaluated with a mirVana RT-qPCR miRNA Detection Kit (Ambion Inc., Austin, TX, USA). The combination of miR-103 and miR-191 was considered an endogenous control and served as a reference in our previous study [29]. TaqMan MicroRNA Assays from Applied Biosystems were used in the PCR system, and the sequences were as follows: miR-182 (Cat. No. 4427975-002334): UUUGGCAAUGGUAGAACUCACACU; miR-103 (Cat. No. 4427975-000439): AGCAGCAUUGUACAGGGCUAUGA; and miR-191 (Cat. No. 4427975-000490): CAACGGAAUCCCAAAAGCAGCU. The expression of miR-182 in the FFPE experiments was computed with the formula 2^-Δcq^.

### Literature

The keywords were used to search the literature of miR-182-5p in LUSC from PubMed, Wiley Online Library, EBSCO, Cochrane Central Register of Controlled Trials, Web of Science, Google Scholar, Ovid, EMBASE, and LILACS, until 5 October 2017, and the keywords were as follows: (cancer OR carcinoma OR adenocarcinoma OR tumour OR tumor OR malignanc* OR neoplas*) AND (Lung OR pulmonary OR respiratory OR respiration OR aspiration OR bronchi OR bronchioles OR alveoli OR pneumocytes OR “air way”) AND (miR-182 OR miRNA-182 OR microRNA-182 OR miR182 OR miRNA182 OR microRNA182 OR “miR 182” OR “miRNA 182” OR “microRNA 182”OR miR-182-5p OR miRNA-182-5p OR microRNA-182-5p). The studies which were included need to meet the following criteria: (1) the expression of miR-182-5p in LUSC must be detected by *Homo sapiens*, and (2) the data of the expression of miR-182-5p can be extracted in the studies.

### Meta-analysis

A comprehensive meta-analysis was performed using Stata 14.0 software by combining the four sources (RT-qPCR data, TCGA data, GEO datasets, and the literature) reporting miR-182 expression in LUSC. The respective meta-analysis for RT-qPCR data, TCGA data, and GEO datasets was also performed. Pooled data in the meta-analysis were assessed by the standard mean difference (SMD) with a 95% confidential interval (CI). Heterogeneity among the eligible microarrays was evaluated by the chi-squared and *I*-squared tests. The effect model was then determined according to the heterogeneity. Specifically, a fixed effects model was conducted for the meta-analysis when the heterogeneity was low (*I*^2^ ≤ 50% and *p* > 0.05) and a random effects model was selected if apparent heterogeneity existed (*I*^2^ > 50% or *p* ≤ 0.05) [30]. A summary receiver operating characteristic (sROC) curve was constructed to describe the diagnostic ability of miR-182-5p in LUSC.

### MiR-182-5p predicted target genes

MiR-182 target genes were projected in silico with 12 databases (miRWalk, Microt4, miRanda, mirbridge, miRDB, miRMap, miRNAMap, Pictar2, PITA, RNAhybrid, Targetscan, and RNA22). Genes present in at least five databases were further regarded as predicted target genes of miR-182. Two databases (Tarbase and miRTarbase) were employed to gather miR-182 target genes with “strong evidence.” All miR-182 target genes verified by western blot, qPCR, or luciferase reporter assays were selected as validated genes. Moreover, we identified weakly expressed genes in LUSC from TCGA database. Finally, target genes of miR-182 were achieved from the three analyses (predicted genes, validated genes, and genes from TCGA database), which were utilized for further gene pathway analysis, GO analysis, statistical analysis, and generating ROC curves. A correlation analysis between hub genes and miR-182 was also conducted. For all analyses described above, a *p*-value < 0.05 was regarded to present a significant difference.

### Functional enrichment analysis via bioinformatics

Predicted target genes were subjected to GO analysis in the DAVID database [31]. The BINGO plugin of Cytoscape was applied to visualize the GO network. The PPI networks were constructed using STRING 10.0 [32]. We also mapped genes to the KEGG database to identify significant signaling pathways. A *p* value < 0.001 was regarded to show statistical significance.

### Statistical analysis

All statistical analyses were conducted using GraphPad 5.0 software. Student’s *t* test was used to detect a significant difference in the miR-182 expression between two groups, and one-way analysis of variance was used to study the miR-182 level among three or more groups. Furthermore, ROC curves were constructed, and the area under the curve (AUC) was calculated to assess the diagnostic role of miR-182 in LUSC. The diagnostic efficacy for LUSC was evaluated as low, moderate, or high depending on the AUC—0.5–0.7 (low), 0.7–0.9 (moderate), and 0.9–1.0 (high). A statistical alteration was considered to occur when *p* < 0.05.

## Results

### Clinical value of miR-182-5p

#### Expression of miR-182-5p in LUSC from TCGA database

**Fig. 1:**
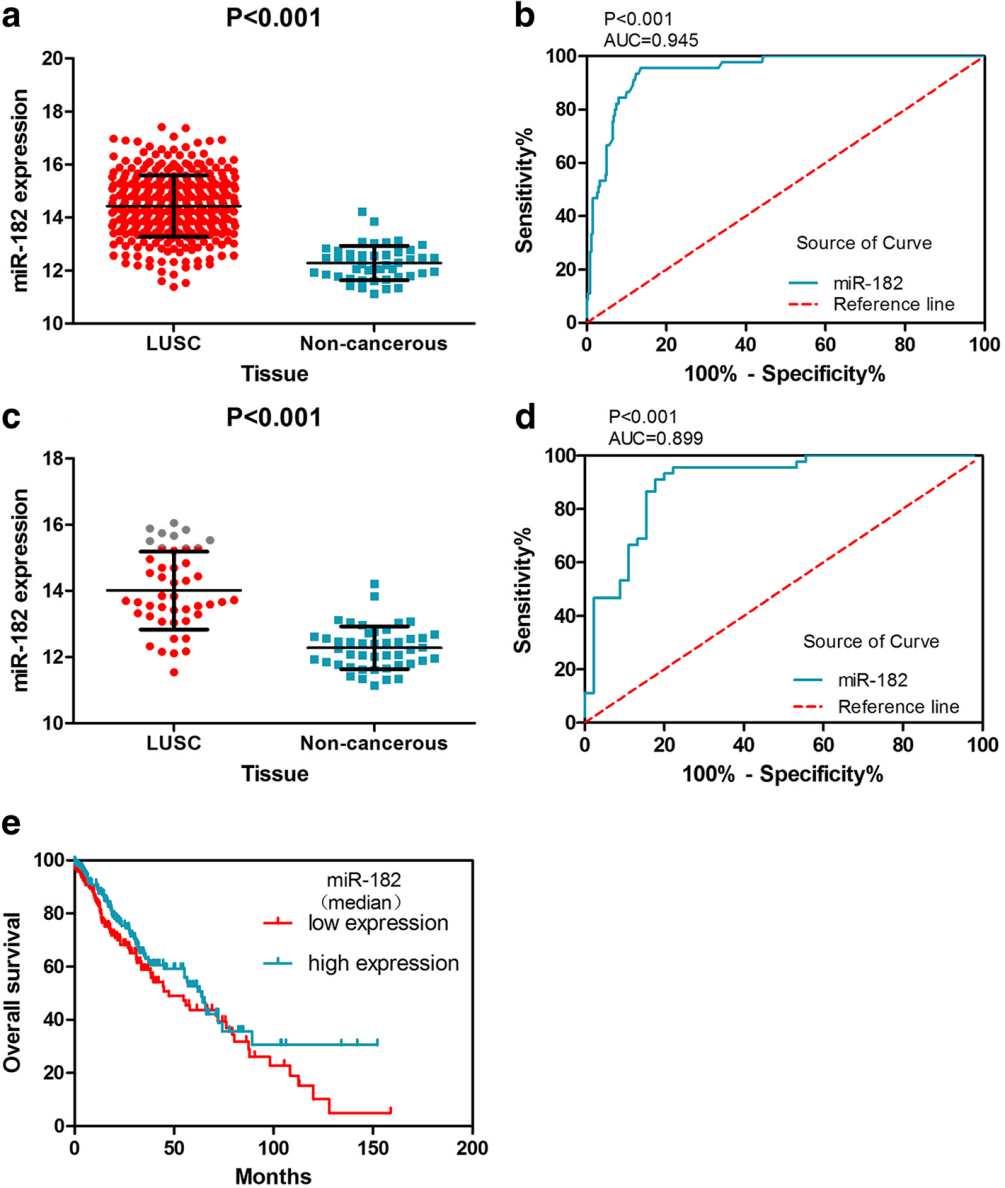
MiR-182 expression is increased in TCGA LUSC samples and has diagnostic value. **a** The expression of miR-182 in 338 LUSC and 45 non-cancerous lung tissues. **b** The ROC curve was generated to assess the diagnostic ability of miR-182 in 338 LUSC and 45 non-cancerous lung tissues. The AUC was 0.945 (95% CI 0.9167 to 0.9728, *p* < 0.001). The sensitivity was 86.39%, and the specificity was 95.56%. **c** MiR-182 expression in LUSC and adjacent normal tissues from 45 LUSC patients. **d** The ROC curve was generated to assess the diagnostic ability of miR-182 in LUSC and adjacent normal tissues from 45 LUSC patients. The AUC was 0.899 (95% CI 0.8329 to 0.9656, *p* < 0.001). The sensitivity was 80.00%, and the specificity was 93.33%. **e** Assessing the prognosis of TCGA LUSC patients using the Kaplan-Meier curve

A total of 338 LUSC cases and 45 adjacent non-cancer cases were collected from TCGA database (Table 2). The expression value of miR-182-5p in the LUSC group was 14.4295 ± 1.16110 and that in the non-cancer group was 12.2828 ± 0.64852. MiR-182-5p expression was clearly over-expressed in the LUSC group in comparison with the non-cancerous group (Fig. 1a). As shown in Fig. 1b, the ROC curve assessed the diagnostic ability of miR-182. To verify this result, we matched the data of 45 patients from TCGA database (Fig. 1c, d). MiR-182 expression was higher in LUSC tissue than in adjacent normal tissues (14.0102 ± 1.17344 and 12.2828 ± 0.64852, respectively, *p* < 0.001).

**Table 2:** Clinicopathological parameters and miR-182 expression in LUSC data from TCGA database

Characteristic		*n*	Relevant expression of miR-182 (log_2_x)		
			Mean ± SD	*t*/*F* value	*p* value
Tissue	LUSC	338	14.4295 ± 1.16110	− 18.590^a^	**< 0.001**
	Non-cancerous	45	12.2828 ± 0.64852		
Gender	Male	254	14.4792 ± 1.14885	1.371	0.171
	Female	84	14.2791 ± 1.19174		
Age (years)	≤ 60	177	14.4281 ± 1.13491	− 0.023	0.982
	> 60	161	14.4310 ± 1.19279		
Pathologic T	T1	80	14.4445 ± 1.13309	0.912^b^	0.435
	T2	189	14.4866 ± 1.16154		
	T3	58	14.3072 ± 1.22759		
	T4	11	13.9834 ± 0.98341		
T	T1 + T2	269	14.4741 ± 1.15120	1.396	0.164
	T3 + T4	69	14.2556 ± 1.19150		
Nodes	No	217	14.4176 ± 1.15198	− 0.250	0.803
	Yes	121	14.4507 ± 1.18181		
Metastasis	No	259	14.4528 ± 1.18483	0.669	0.504
	Yes	79	14.3529 ± 1.08334		
Pathologic stage	I	157	14.3819 ± 1.16704	0.654^b^	0.581
	II	125	14.4825 ± 1.20539		
	III	50	14.4849 ± 1.05204		
	IV	3	13.6550 ± 0.94917		
Stage	I-II	282	14.4265 ± 1.18313	− 0.065	0.948
	III-IV	53	14.4379 ± 1.05597		
Anatomic organ subdivision	L_lower	41	14.3997 ± 1.31582	0.795^b^	0.529
	L_upper	90	14.2951 ± 1.00576		
	R_lower	76	14.5891 ± 1.17043		
	R_middle	11	14.6375 ± 1.30430		
	R_upper	96	14.4124 ± 1.12167		
Tumor location	Peripheral	73	14.3940 ± 1.12931	0.288	0.774
	Central	107	14.3435 ± 1.17386		

Statistically significant results (*p* < 0.05) are indicated in bold

*LUSC* lung squamous cell carcinoma, *SD* standard deviation

^a^Student’s *t* test was used for comparison between the experimental and control groups

^b^One-way analysis of variance (ANOVA) was used for the analysis of five groups

Kaplan-Meier curves (Fig. 1e) were constructed to analyze the prognosis of miR-182-5p in LUSC patients. The curves display the median survival of LUSC patients with high miR-182-5p expression (63.73 months) and those with low miR-182-5p expression (47.43 months).

#### LUSC microarrays from the GEO database

**Fig. 2:**
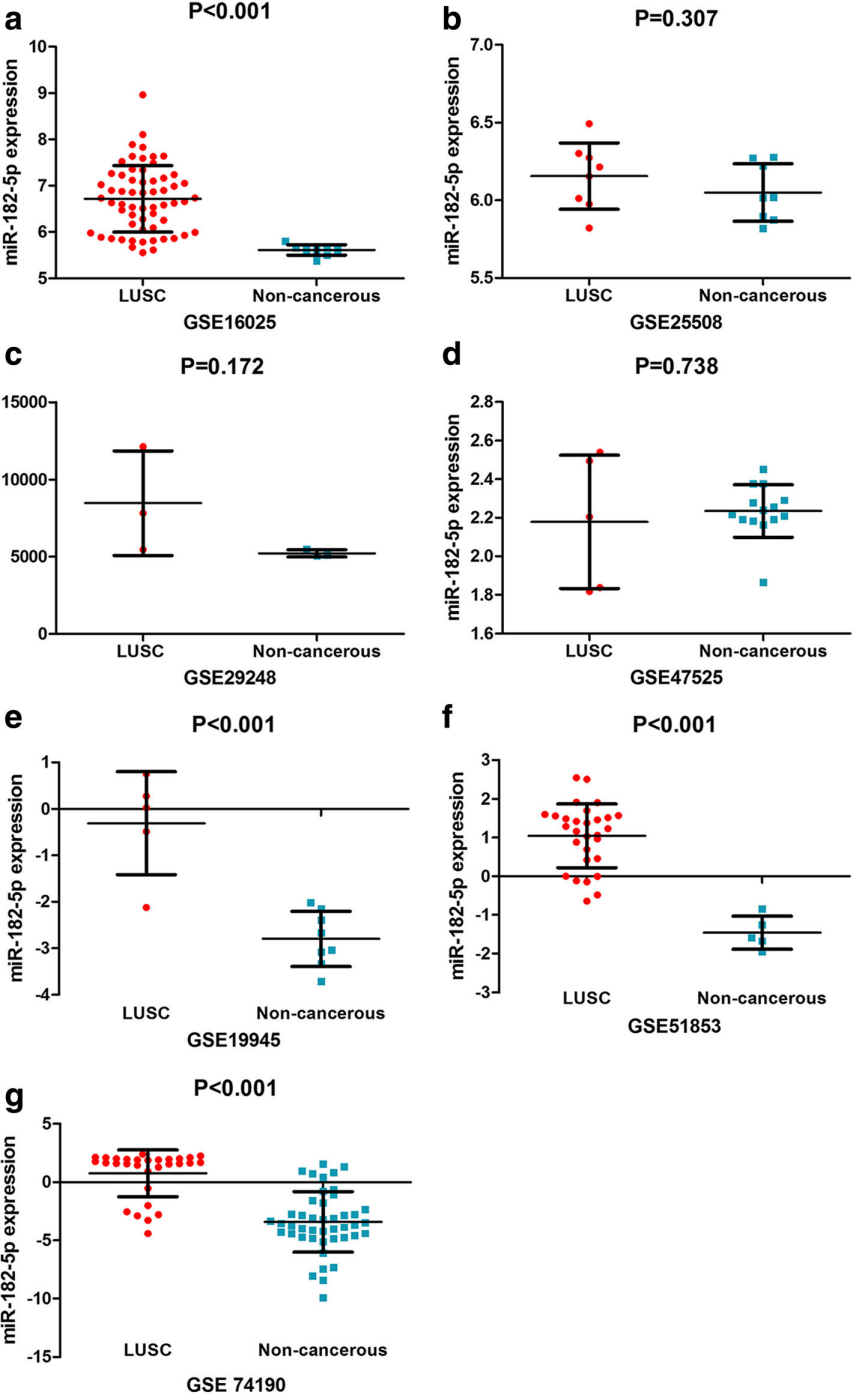
MiR-182-5p expression is over-expressed in LUSC tissues according to GEO microarrays. **a** Microarray GSE16025. **b** Microarray GSE25508. **c** Microarray GSE29248. **d** Microarray 47,525. **e** Microarray GSE19945. **f** Microarray GSE51853. **g** Microarray GSE74190

**Fig. 3:**
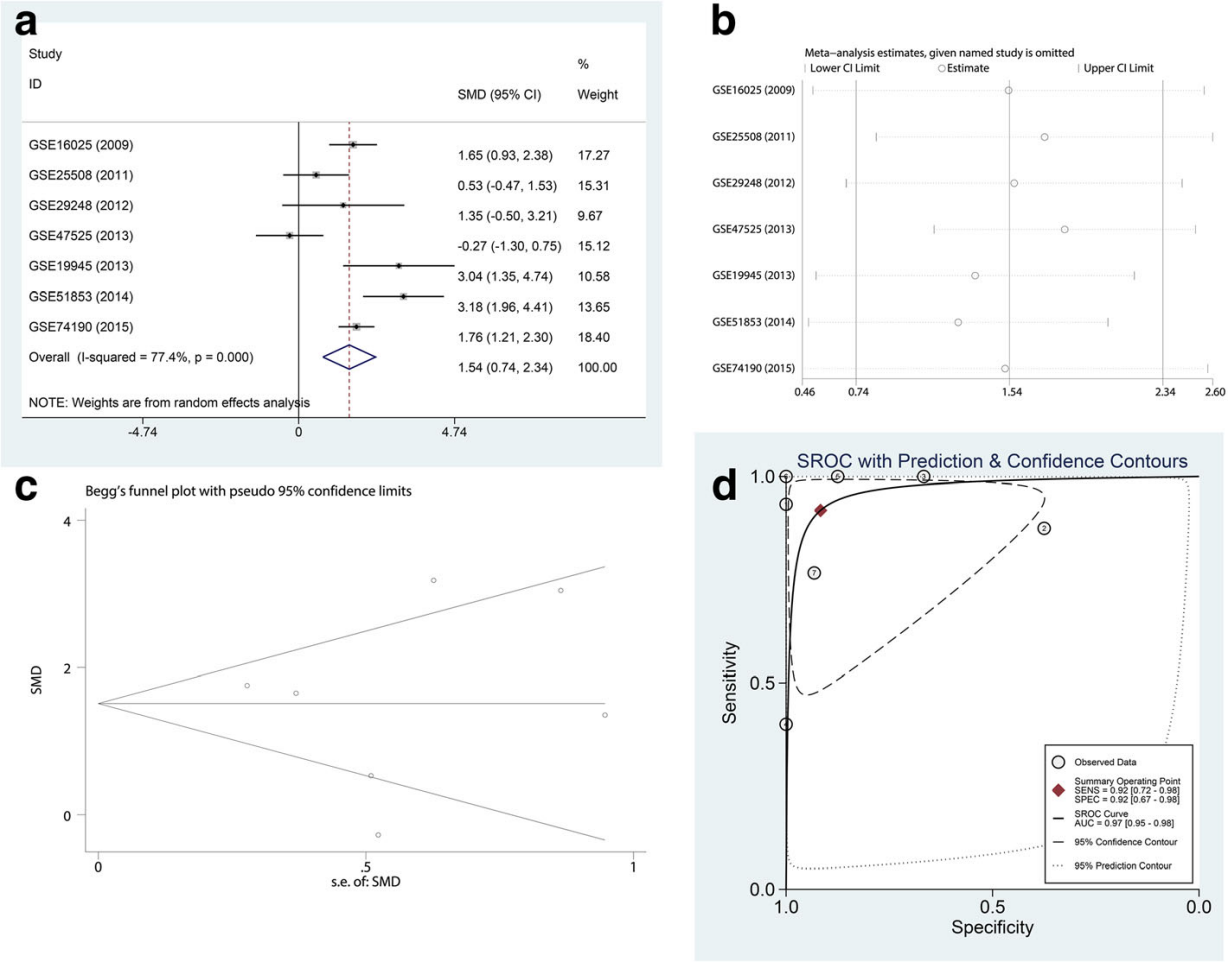
Meta-analysis of GEO microarrays. **a** Forest plot of miR-182-5p expression data from GEO microarrays. The pooled SMD of miR-182-5p was 1.54 (95% CI 0.74 to 2.34) by the random effects model. The I-squared value was 77.4%, and the *p* value was less than 0.001. **b** Sensitivity analysis of GEO microarrays. **c** A funnel plot was used to show the publication bias of GEO microarrays (Begg’s method). **d** Summary receiver operating characteristic (sROC) curve (AUC) of miR-182-5p in the diagnosis of LUSC data from the GEO microarrays. The AUC was 0.97 (95% CI 0.95–0.98)

GEO microarrays can be regarded as an auxiliary means to validate the expression of miR-182-5p in LUSC. A total of seven microarrays were selected from the GEO database, namely, GSE16025, GSE25508, GSE29248, GSE47525, GSE19945, GSE51853, and GSE74190 (Fig. 2). Four microarrays (GSE16025, GSE19945, GSE51853, and GSE74190) showed statistical significance in which the miR-182-5p expression level was remarkably increased in LUSC tissues. The expression of miR-182-5p in the GEO microarrays is shown in Table 3. The meta-analysis results are shown in Fig. 3. The forest plot (Fig. 3a) included the miR-182-5p expression data from the seven microarrays. The pooled SMD of miR-182-5p was 1.54 (95% CI 0.74 to 2.34) by the random effects model. The I-squared value was 77.4%, and the *p* value was less than 0.001. Furthermore, the sensitivity analysis (Fig. 3b) indicated no significant difference among the microarrays. We also assessed the publication bias using a funnel plot (Fig. 3c). The *p* value from Begg’s test was 1.000 and that from Egger’s test was 0.939. The sROC curve of the GEO microarrays is shown in Fig. 3d. The AUC was 0.97 (95% CI 0.95–0.98). Based on these results, we conclude that these microarrays had no significant publication bias.

**Table 3:** Expression of miR-182-5p in the GEO microarrays

ID	Publication year	Tissue	*n*	Mean ± SD	*t* value	*p* value
GSE16025	2009	LUSC	60	6.718668 ± 0.7187121	11.157^a^	**< 0.001**
		Non-cancerous	10	5.60987 ± 0.1125845		
GSE25508	2011	LUSC	8	6.1557 ± 0.21346	1.060	0.307
		Non-cancerous	8	6.0497 ± 0.18564		
GSE29248	2012	LUSC	3	8467.2363 ± 3393.2628	1.659	0.172
		Non-cancerous	3	5209.7237 ± 234.24406		
GSE47525	2013	LUSC	5	2.1787 ± 0.34594	− 0.356	0.738
		Non-cancerous	14	2.2353 ± 0.13647		
GSE19945	2013	LUSC	5	− 0.309311 ± 1.1072303	5.341	**< 0.001**
		Non-cancerous	8	− 2.79921 ± 0.5918523		
GSE51853	2014	LUSC	29	1.048088 ± 0.8260201	6.576	**< 0.001**
		Non-cancerous	5	− 1.458859 ± 0.4262417		
GSE74190	2015	LUSC	30	0.7706 ± 2.0075	7.417	**< 0.001**
		Non-cancerous	44	− 3.4043 ± 2.5973		

Statistically significant results (*p* < 0.05) are indicated in bold

*LUSC* lung squamous cell carcinoma, *SD* standard deviation

^a^Student’s *t* test was used for comparison between the experimental and control groups

#### RT-qPCR analysis

**Fig. 4:**
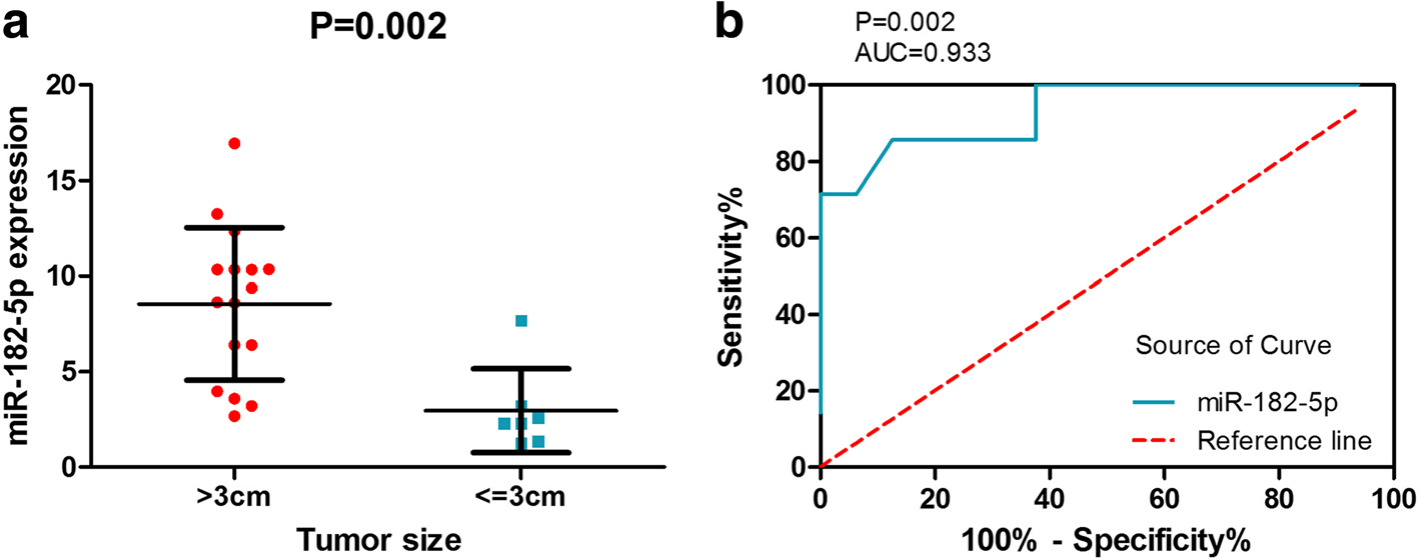
Diagnostic value and expression of miR-182-5p in LUSC. **a** MiR-182-5p expression in patients whose tumor size was greater than 3 cm and in patients whose tumor size was less than or equal to 3 cm. **b** The ROC curve was generated to assess the diagnostic ability of miR-182-5p in tumor size. The AUC was 0.933 (95% CI 0.8206 to 1.045, *p* = 0.002). The sensitivity was 85.71%, and the specificity was 87.50%

We detected the clinical expression level of miR-182-5p by RT-qPCR in 23 LUSC and 23 non-cancerous lung tissues. The miR-182-5p expression whose tumor size was greater than 3 cm was 8.55 ± 3.99, and the expression of whose tumor size was less than 3 cm was 2.96 ± 2.20 (Fig. 4a). In Fig. 4b, the ROC curves show the diagnostic value of miR-182-5p in tumor size.

#### Literature

According to the inclusion and exclusion criteria, a total of eight articles examined both LUSC and miR-182-5p [21, 33–39]. However, these articles reported the *p* values of miR-182-5p expression rather than the mean and standard deviation. Therefore, no data could be extracted.

#### Meta-analysis of TCGA, GEO, PCR, and literature analyses

**Fig. 5:**
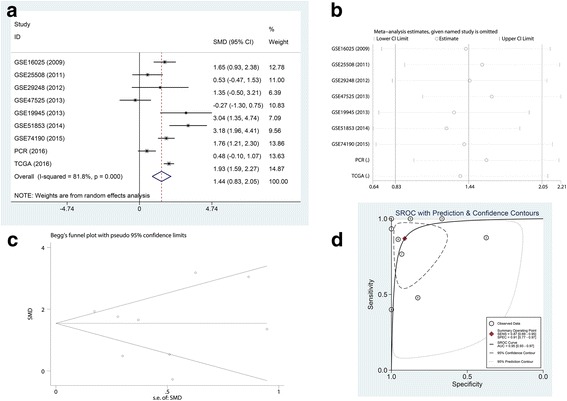
Meta-analysis of PCR, TCGA database, and GEO microarrays. **a** Forest plot of miR-182-5p expression data from PCR, TCGA database, and GEO microarrays. The pooled SMD of miR-182-5p was 1.44 (95% CI 0.83 to 2.05) by the random effects model. The I-squared value was 81.8%, and the *p* value was less than 0.001. **b** Sensitivity analysis of PCR, TCGA database, and GEO microarrays. **c** The funnel plot shows the publication bias of PCR, TCGA database, and GEO microarrays (Begg’s method). **d** sROC curve (AUC) of miR-182-5p in the diagnosis of LUSC data from PCR, TCGA database, and GEO microarrays. The AUC was 0.95 (95% CI 0.93–0.97)

We performed a comprehensive meta-analysis using data from TCGA database, GEO microarrays, and PCR. Regarding the literature, the data could not be extracted. A total of 501 LUSC cases and 148 non-cancerous cases were extracted. The random-effect was used in the meta-analysis because the I-squared value was 81.8%. The I-squared value may be caused by the differences in patients, samples processing methods, and statistical methods. The forest plot (Fig. 5a) included the miR-182-5p expression data from PCR, TCGA database, and GEO microarrays. The pooled SMD of miR-182-5p was 1.44 (95% CI 0.83 to 2.05) using the random effects model. The I-squared value was 81.8%, and the *p* value was less than 0.001. The sensitivity analysis (Fig. 5b) indicated no significant difference among studies. The funnel plot (Fig. 5c) showed a publication bias among these studies. The *p* value obtained from Begg’s test was 0.754 and that from Egger’s test was 0.678. The sROC curve is shown in Fig. 5d. The AUC was 0.95 (95% CI 0.93–0.97). In summary, these studies showed a mild publication bias.

### Molecular mechanism of miR-182-5p

#### Prediction of miR-182-5p target genes

**Fig. 6:**
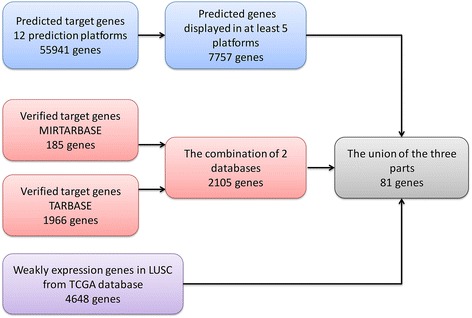
Process of screening target genes

The prediction of miR-182-5p target genes was performed using 12 gene prediction platforms. We chose the predicted genes displayed in at least five platforms, which was 7757. The number of verified target genes was 2105. We next downloaded 4648 genes with low miR-182-5p expression in LUSC from TCGA database. Finally, we calculated the union of the three groups, and a total of 81 target genes were chosen. The screening process is displayed in Fig. 6.

#### GO and KEGG analyses

**Fig. 7:**
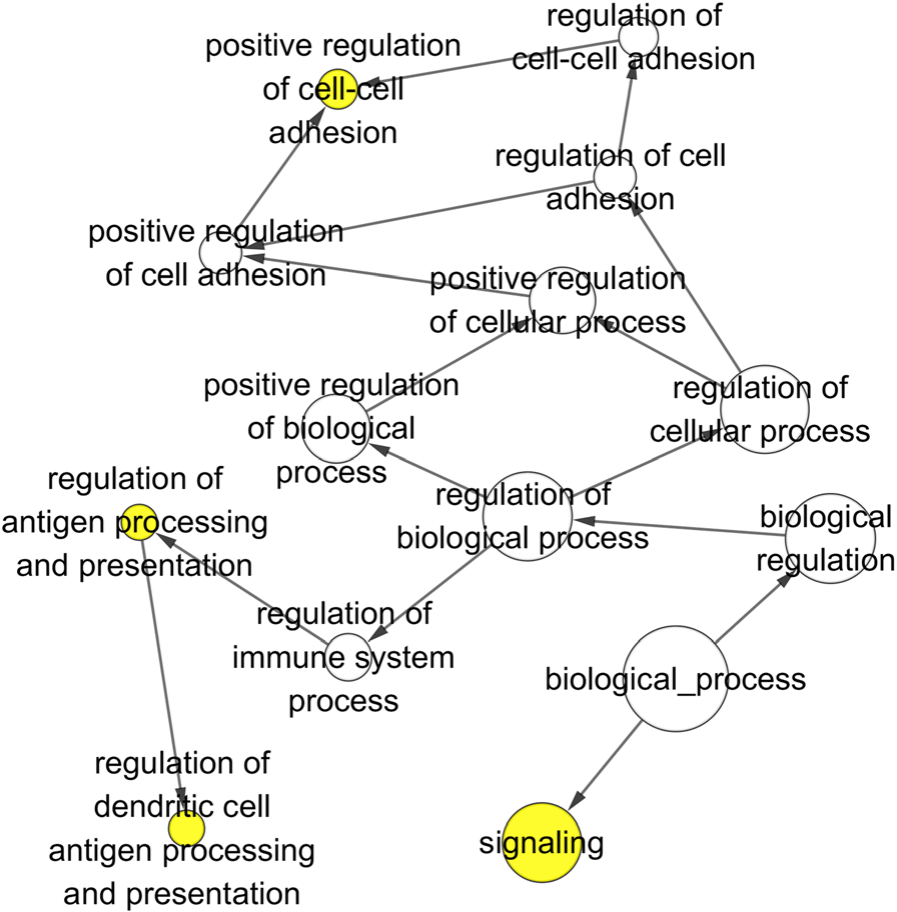
GO biological process (GO-BP) network for the predicted target genes. Nodes represent GO items. Yellow nodes imply that the items are statistically significant (*p* < 0.01). White nodes imply that the items only take part in connecting items but are not statistically significant

**Fig. 8:**
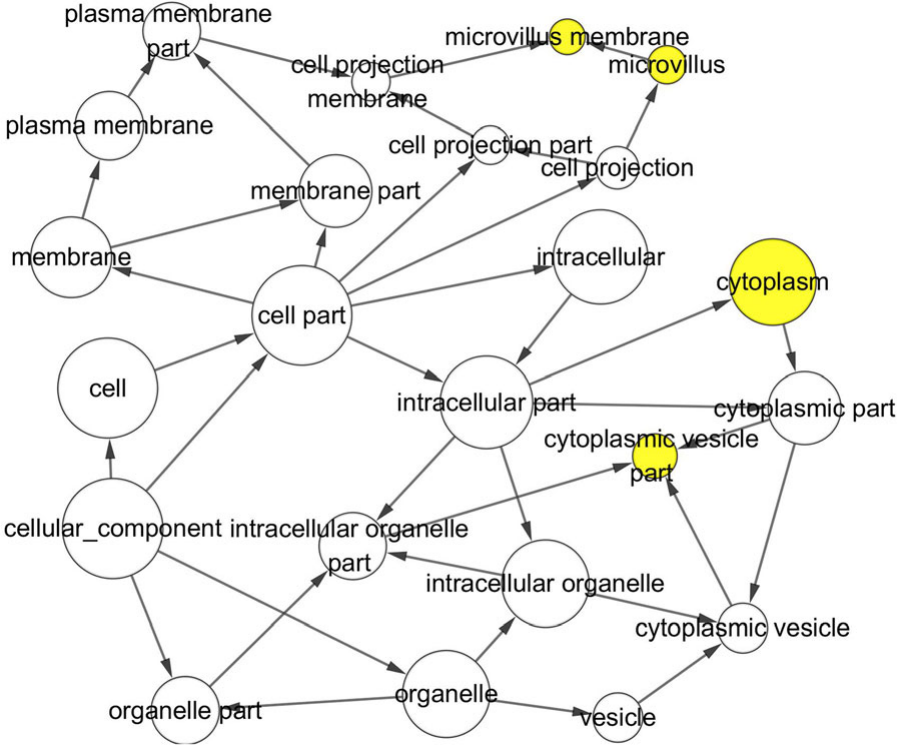
GO cellular component (GO-CC) network for the predicted target genes. Nodes represent GO items. Yellow nodes imply that the items are statistically significant (*p* < 0.01). White nodes imply that the items only take part in connecting items but are not statistically significant

**Fig. 9:**
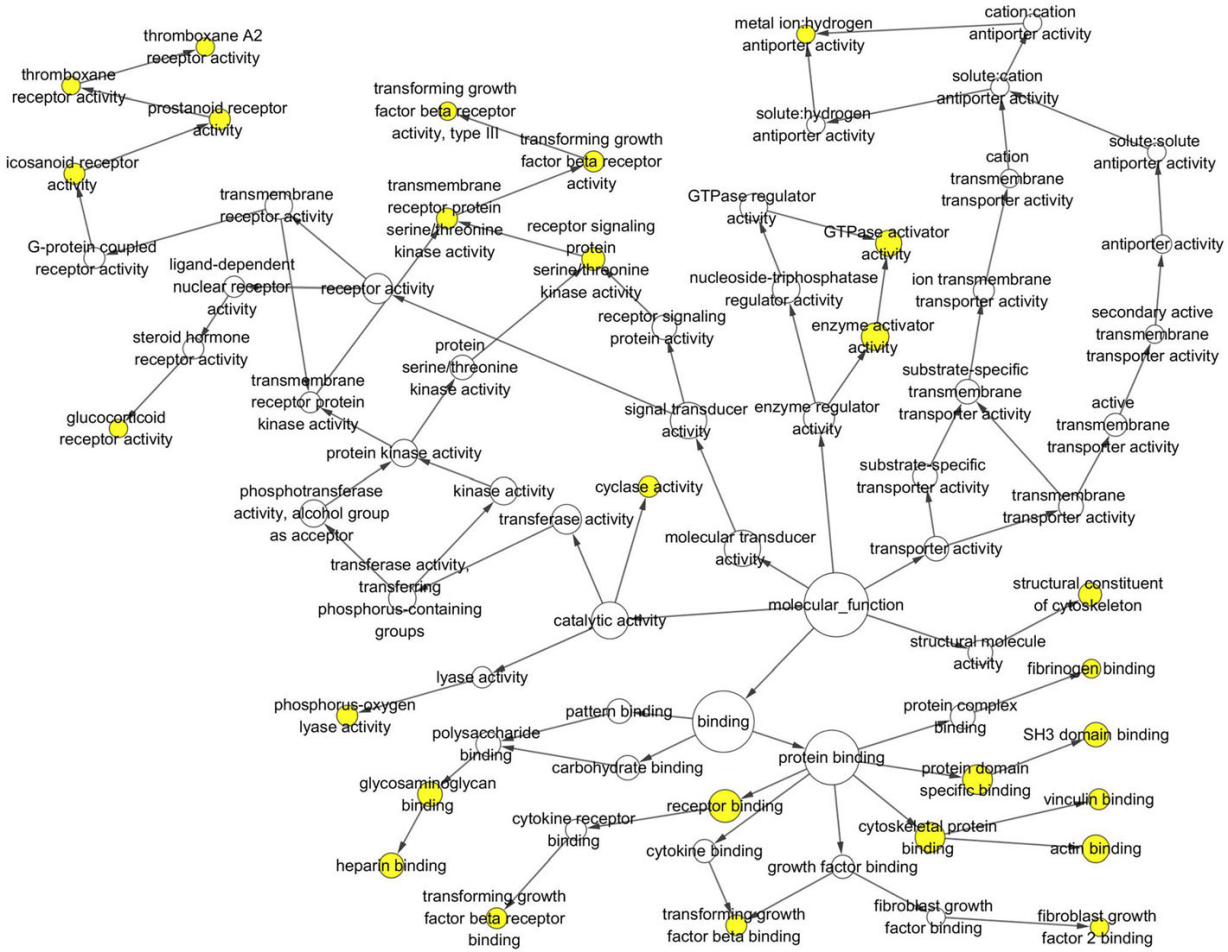
GO molecular function (GO-MF) network for the predicted target genes. Nodes represent GO items. Yellow nodes imply that the items are statistically significant (*p* < 0.05). White nodes imply that the items only take part in connecting items but are not statistically significant

Table 4 shows part of the GO enrichment and KEGG pathway analysis results of 81 target genes by DAVID (https://david.ncifcrf.gov/). The GO enrichment analysis was composed of three parts: GO biological process (GO-BP), GO cellular component (GO-CC), and GO molecular function (GO-MF). GO-BP included 56 items, the most important of which were positive regulation of transforming growth factor beta receptor signaling pathway and ventricular septum morphogenesis. GO-CC included 17 items, the most important of which were extracellular exosome and an extrinsic component of the membrane. GO-MF included 15 items, and the target genes were largely involved in protein binding and SH3 domain binding. With respect to the KEGG pathway analysis, the results included nine items. Among these pathways, the Rap1 signaling pathway and platelet activation were important. We also show the GO network for the predicted target genes in Figs. 7, 8, and 9. One node represents one term. Yellow nodes indicate that the terms are more significant.

**Table 4:** Enriched GO and KEGG items

Category	Item	Count
GO-BP	Positive regulation of transforming growth factor beta receptor signaling pathway	4
	Ventricular septum morphogenesis	4
	Positive regulation of early endosome to late endosome transport	3
	Positive regulation of gene expression	7
	Cellular response to prostaglandin E stimulus	3
	Signal transduction	13
	Cell migration	5
	Negative regulation of cell migration	4
GO-CC	Extracellular exosome	23
	Extrinsic component of membrane	4
	Plasma membrane	29
	Apical plasma membrane	6
	Cell periphery	3
	Cytosol	23
	Cytoplasm	32
	Focal adhesion	6
GO-MF	Protein binding	49
	SH3 domain binding	4
	Transcriptional activator activity, RNA polymerase II core promoter proximal region sequence-specific binding	5
	Heparin binding	4
	Actin binding	5
	GTPase activator activity	5
	Vinculin binding	2
	Activin binding	2
KEGG	Rap1 signaling pathway	5
	Platelet activation	4
	MicroRNAs in cancer	5
	cGMP-PKG signaling pathway	4
	TGF-beta signaling pathway	3
	Salivary secretion	3
	Pathways in cancer	5
	Vascular smooth muscle contraction	3

Table shows eight items each from GO-BP, GO-CC, GO-MF and KEGG

#### PPI network of target genes

**Fig. 10:**
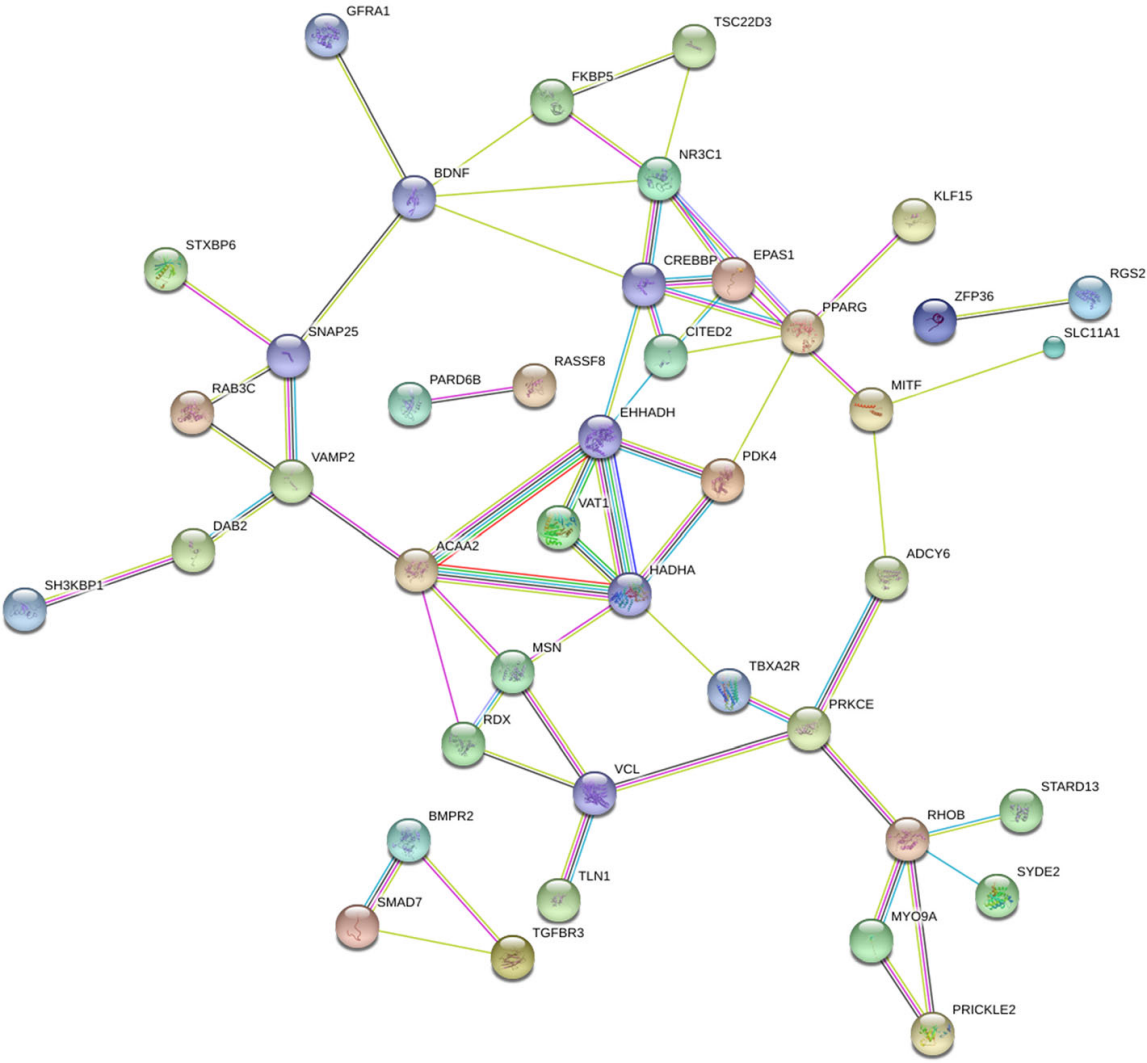
Center genes from the STRING protein-protein interaction network. Nodes represent gene-encoded proteins. Connections between nodes represent the relationship between proteins. A bold line implies a higher confidence level

We identified 31 proteins in the PPI network (Fig. 10), some of which were not associated with other proteins. The more connections between proteins indicate that the protein is more important in LUSC. According to the PPI network, EPAS1, PRKCE, NR3C1, and RHOB are hub genes in LUSC.

#### Clinical expression of hub genes

**Fig. 11:**
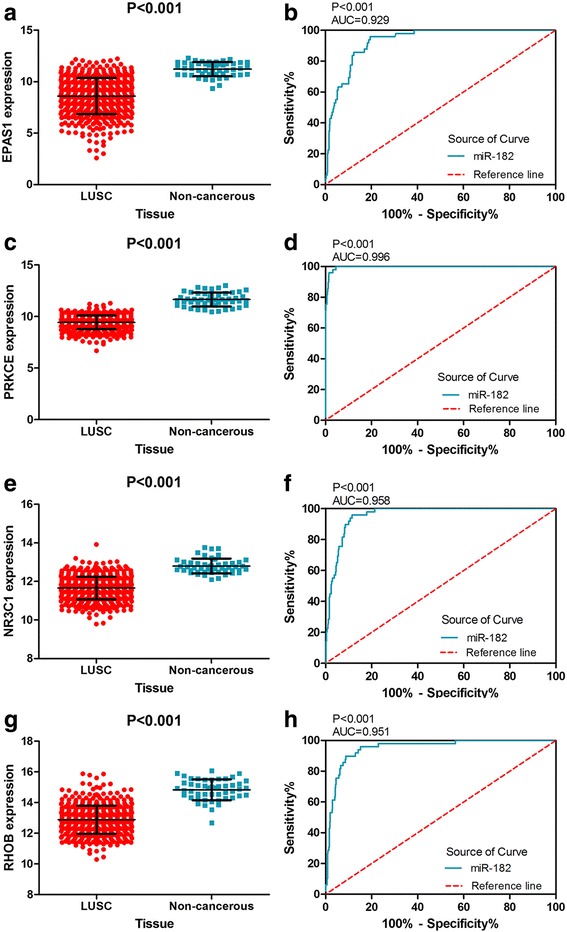
The expression of four hub genes was decreased in TCGA LUSC samples and ROC curve analysis. **a** The expression of EPAS1 in 502 LUSC and 49 non-cancerous lung tissues. **b** ROC curve was generated to assess the diagnostic ability of EPAS1 in 502 LUSC and 49 non-cancerous lung tissues. The AUC was 0.929 (95% CI 0.9023 to 0.9558, *p* < 0.001). **c** The expression of PRKCE in 502 LUSC and 49 non-cancerous lung tissues. **d** The ROC curve was generated to assess the diagnostic ability of PRKCE in 502 LUSC and 49 non-cancerous lung tissues. The AUC was 0.996 (95% CI 0.9929 to 0.9995, *p* < 0.001). **e** The expression of NR3C1 in 502 LUSC and 49 non-cancerous lung tissues. **f** The ROC curve was generated to assess the diagnostic ability of NR3C1 in 502 LUSC and 49 non-cancerous lung tissues. The AUC was 0.958 (95% CI 0.9404 to 0.9749, *p* < 0.001). **g** The expression of RHOB in 502 LUSC and 49 non-cancerous lung tissues. **h** The ROC curve was generated to assess the diagnostic ability of RHOB in 502 LUSC and 49 non-cancerous lung tissues. The AUC was 0.929 (95% CI 0.9238 to 0.9774, *p* < 0.001)

**Fig. 12:**
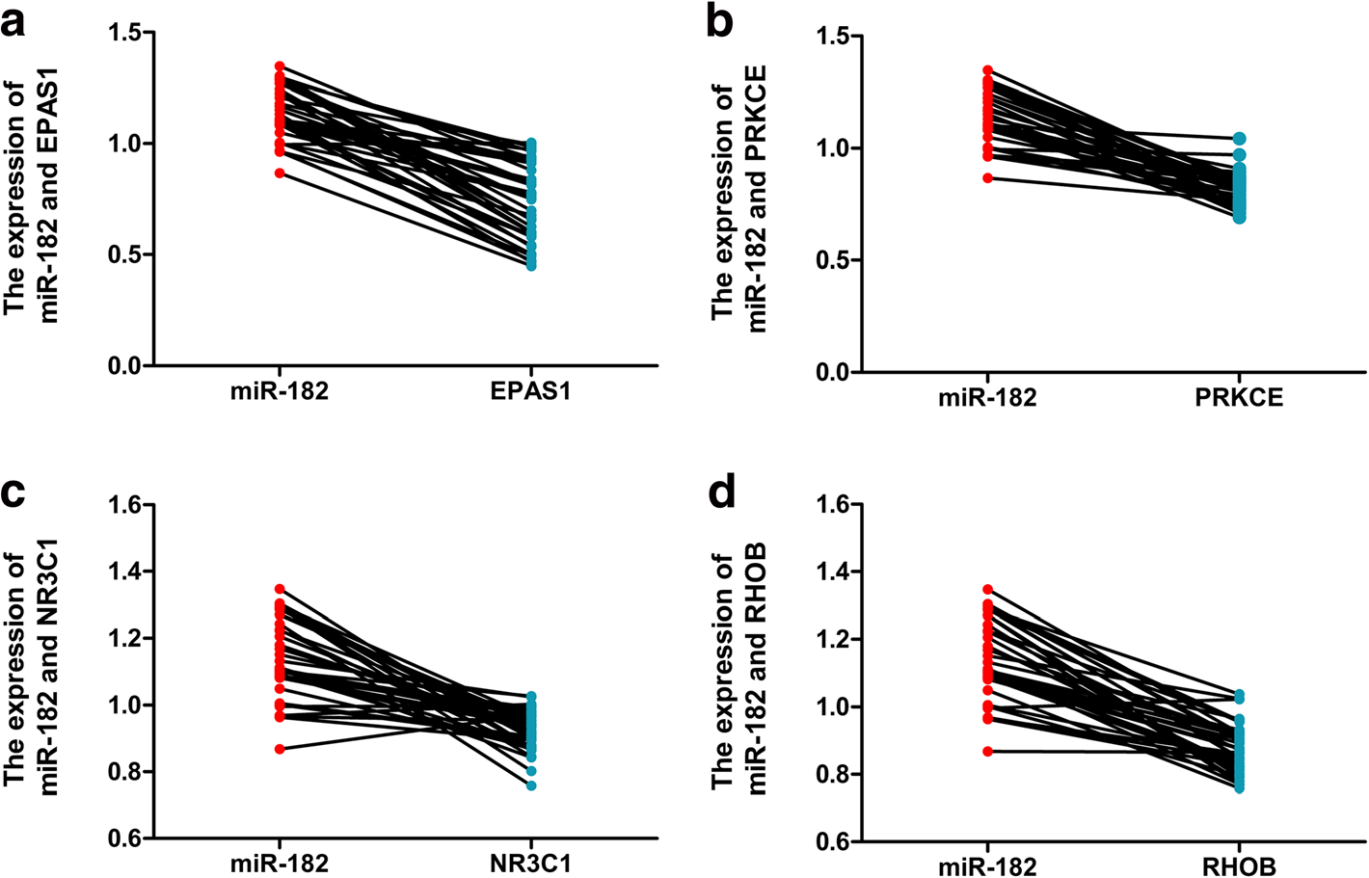
Correlation analysis of the four hub genes decreased in 38 paired LUSC samples from TCGA database. **a** Correlation between miR-182 and EPAS1. **b** Correlation between miR-182 and PRKCE. **c** Correlation between miR-182 and NR3C1. **d** Correlation between miR-182 and RHOB

Among the 81 target genes, EPAS1, PRKCE, NR3C1, and RHOB were located in the center of the PPI network. There were more connections between these four genes, which may indicate that these genes contribute to LUSC. We chose four hub genes (EPAS1, PRKCE, NR3C1, and RHOB) to analyze their clinical expression in 502 LUSC and 49 non-cancerous cases from TCGA database. The expression of EPAS1, PRKCE, NR3C1, and RHOB was decreased in LUSC (Table 5). Figure 11a, c, e, g shows the expression of the four hub genes in LUSC and non-cancerous tissues. Figure 11b, d, f, h shows the ROC curves of the diagnostic ability of the four genes. The AUCs were 0.929 (95% CI 0.9023 to 0.9558, *p* < 0.001), 0.996 (95% CI 0.9929 to 0.9995, *p* < 0.001), 0.958 (95% CI 0.9404 to 0.9749, *p* < 0.001), and 0.929 (95% CI 0.9238 to 0.9774, *p* < 0.001), respectively. Correlations between the four hub genes and miR-182-5p expression are shown in Fig. 12. The expression of the four hub genes was significantly negatively related to miR-182-5p expression in LUSC.

**Table 5:** Expression of four hub genes in LUSC data from TCGA database

Gene	Mean ± SD		t	*p* value
	LUSC	Non-cancerous		
EPAS1	8.606392 ± 1.7655376	11.220022 ± 0.6834735	− 20.831	**< 0.001**
PRKCE	9.428288 ± 0.6642029	11.656338 ± 0.6706216	− 22.394	**< 0.001**
NR3C1	11.654198 ± 0.5869350	12.798826 ± 0.3824635	− 18.890	**< 0.001**
RHOB	12.884868 ± 0.9119690	14.829378 ± 0.6793070	− 18.478	**< 0.001**

Statistically significant results (*p* < 0.05) are indicated in bold

*LUSC* lung squamous cell carcinoma, *SD* standard deviation

## Discussion

At present, LUSC is one of the most common cancers and is the chief cause of cancer deaths [1, 40]. Misdiagnosis or metastasis can increase the mortality rate. Therefore, miRs including miR-182-5p are regarded as a new tool used to diagnose LUSC [41].

In our study, we gathered a large amount of data on miR-182-5p expression in LUSC from TCGA and GEO databases and analyzed data from 23 paired clinical LUSC tissues. Herein, a meta-analysis was performed to explore the clinical value of miR-182-5p in LUSC.

There were 338 LUSC cases and 45 adjacent non-cancer cases in TCGA database. The data from TCGA database showed that the miR-182-5p expression in LUSC tissues was higher than in adjacent normal tissues, which indicated that miR-182-5p expression was associated with LUSC. We also included seven microarrays (GSE16025, GSE25508, GSE29248, GSE47525, GSE19945, GSE51853, and GSE74190) in the GEO database. In addition to GSE47525, other microarrays showed an increasing trend in miR-182-5p expression in LUSC compared to non-cancerous tissues. Among them, four microarrays (GSE16025, GSE19945, GSE51853, and GSE74190) showed statistical significance. However, in GSE47525, the result was opposite. MiR-182-5p expression was lower in LUSC tissue than in non-cancerous tissue. The result of GSE47525 may be caused by the small number of patient samples. According to RT-qPCR, miR-182-5p expression was correlated with tumor size. The expression of miR-182-5p tended to be higher when the tumor size was greater than 3 cm. As the tumor is growing, the expression of miR-182-5p was also increasing. The result revealed that the miR-182-5p was important in the progress of LUSC, and miR-182-5p could indicate the deterioration of LUSC. On the basis of the result, miR-182-5p can provide a biomarker to detect the occurrence and development of LUSC. The meta-analysis, which included data from TCGA database, the GEO database, RT-qPCR, and the literature, was the highlight of our study. The meta-analysis rendered the most comprehensive data on miR-182-5p. The pooled SMD of miR-182-5p was 1.44 (95% CI 0.83 to 2.05) by the random effects model, which showed that the high miR-182-5p expression in LUSC was consistent with the literature [8, 13, 14, 35, 39]. Therefore, we conclude that miR-182-5p is markedly over-expressed in LUSC, consistent with the existing research. And the results showed an obvious relationship between the miR-182-5p expression and LUSC.

We also predicted miR-182-5p target genes using 12 prediction platforms and performed a bioinformatics analysis by GO enrichment, KEGG pathway, and PPI network analyses. The GO enrichment and KEGG pathway analyses included 97 items. In GO-BP, the pathway of apoptotic process included the target genes PRKCE, NR3C1, and RHOB. However, the pathway of apoptotic process in LUSC is still unclear. In GO-CC, the cytosol and cytoplasm were enriched in four hub genes. But there was no study of the relationship between the pathway and LUSC. As for GO-MF, EPSA1, PRKCE, NE3C1, and RHOB were all involved in SH3 domain binding. Shim et al. found that SH3 domain-binding protein 1 could suppress the growth of LUSC [42]. Through the thinking, we can slow down the progress of LUSC by SH3 domain binding pathway. Additionally, the KEGG pathway analysis revealed that PRKCE is involved in the pathway of MicroRNAs in cancer, the cGMP-PKG signaling pathway, and pathway of vascular smooth muscle contraction. The function of these pathways in LUSC remains to be studied.

According to our bioinformatics analysis, four genes (EPAS1, PRKCE, NR3C1, and RHOB) were regarded as hub genes in LUSC. EPAS1, which is also known as hypoxia-inducible factor-2α (HIF-2α), belongs to the family of hypoxia-inducible factors (HIFs) [43]. In our study, the expression of EPAS1 was negatively correlated with the expression of miR-182-5p in LUSC. In LUSC, EPAS1 plays the role of a HIF [44]. According to recent studies, the high level of EPAS1 expression could lead to a poor prognosis by increasing the tumor size and angiogenesis [43, 45, 46]. These findings are consistent with the conclusions of our current study.

PRKCE, which consists of 32 exons, is a member of the protein kinase C (PKC) family and regulates the formation of protein kinase C epsilon type (PKCε) [47]. According to our statistical analysis, the high miR-182-5p expression in LUSC is accompanied by the low expression of PRKCE. As an enzyme, PKCε influences many cellular functions, such as growth, division, and transcription factor regulation [48–50]. Wang et al. [51] discovered that PKCε is oncogenic and associated with the occurrence of lung cancer. They also found that PRKCE increases PKCε expression in LUSC.

NR3C1 is also known as GR or GCR and encodes a glucocorticoid receptor to participate in inflammation, cell proliferation, and differentiation [52]. NR3C1 plays an anti-inflammatory role in the development and metastasis of LUSC [53, 54]. Therefore, NR3C1 is important for inhibiting tumor progression.

RHOB belongs to the Ras homolog gene family. RHOB plays a role in cell proliferation and survival [55]. RHOB also inhibits tumor growth. If RHOB is lacking, the tumor frequency increases [56]. A recent study found that the lack of RHOB often occurs in LUSC [57]. According to our study, the expression of RHOB is downregulated in LUSC, consistent with the report by Mazières et al. [56].

According to the present study, miR-182-5p is upregulated in LUSC and plays a pivotal role in the process of LUSC. Through our research, miR-182-5p is found that it is involved in several biological processes to inhibit LUSC progression and improve the cure rate, and it can offer a new idea of LUSC diagnosis and therapy in molecular mechanism to us.

## Conclusion

Our study collected a lot of data from TCGA, GEO, and RT-qPCR and verified the clinical value and diagnostic significance of the high miR-182-5p expression in LUSC. According to the result of target genes, 81 genes were related to the molecular mechanism of miR-182-5p in LUSC. The result of GO and KEGG pathway can provide the idea to cure LUSC in the molecular mechanism.

## Abbreviations

AUC: Area under the curve
CI: Confidential interval
GEO: Gene expression omnibus
GO: Gene ontology
GO-BP: GO biological process
GO-CC: GO cellular component
GO-MF: GO molecular function
KEGG: Kyoto Encyclopedia of Genes and Genomes
LUSC: Lung squamous cell carcinoma
miRNAs: MicroRNAs
NSCLC: Non-small cell lung cancer
PPI: Protein-protein interaction
RT-qPCR: Real-time quantitative polymerase chain reaction
SMD: Standard mean difference
sROC: Summary receiver operating characteristic
TCGA: The Cancer Genome Atlas
